# Beyond montreal: a critical evaluation of the Montreal Criteria for uterine transplantation

**DOI:** 10.1007/s40592-026-00287-0

**Published:** 2026-05-26

**Authors:** Jeffrey Pannekoek

**Affiliations:** Center for Bioethics, Cleveland Clinic, Cleveland, OH, United States

**Keywords:** Uterine transplantation, Gender care, Uterine factor infertility, Montreal Criteria

## Abstract

Uterine transplantation (UTx) is an innovative and exciting development that is aimed at making available the experience of gestation to those for whom it may otherwise be out of reach. Given the personal and social importance of this experience to many people, this is a laudable goal. Outside of at least one historic outlier, this practice has primarily been discussed in the context of resolving uterine factor infertility (UFI) for cisgender women. The guidelines and protocols that have been established around UTx understand Uterine Factor Infertility (UFI) as the indication for UTx. This article critically evaluates the Montreal Criteria (2012, 2013) as the primary ethical guidelines regarding UTx. This evaluation focuses on and challenges the way the Criteria structure the discussion around UFI as indication for UTx and the requirement for female genetics. I argue that, outside of the initial experimental design of UTx, there are prima facie grounds for making UTx available outside of the indication of UFI, and that for reasons both internal and external to the Montreal Criteria’s analysis of UTx, there are no overwriting ethical reasons to limit UTx to cis-gender women.

## Introduction

Uterine transplantation (UTx) is an innovative and exciting development that is aimed at making available the experience of gestation to those for whom it may otherwise be out of reach. Given the personal and social importance of this experience to many people, this is a laudable goal. Outside of at least one historic outlier, this practice has primarily been discussed in the context of resolving uterine factor infertility (UFI) for cis-gender women.^1^ The guidelines and protocols that have been established around UTx understand Uterine Factor Infertility (UFI) as the indication for UTx. UFI as a medical diagnosis requires that the patient either has a uterus that does not function such that it can sustain gestation or that a uterus is absent where it would normally be present. While it can be difficult to account for biological norms, one common sense way of describing the norm for having a uterus is that the person’s biological sex is female. However, this merely defers the issue of categorization to that of biological sex. This merely defers the difficulty, since there are a range of options for expressing the metaphysics of biological sex, none of which are without exception or complication. Positing UFI as the primary or exclusive indication for UTx generally presumes a lot—too much—about the socio-biological factors that contribute to sex and gender identity.

There is at least a tacit recognition of this fact in some of the essential literature on UTx. The article that introduced the *Montreal Criteria*, the primary ethical guidelines on the topic of uterine transplantation, recognizes that what UTx is aimed to resolve is not UFI as such—since there are obviously people with UFI who do not wish to experience (further) gestation. Rather, UTx is aimed at providing the experience of gestation to those for whom this experience is sufficiently valuable and beneficial such that the risks of transplantation are outweighed by those benefits. It's not evident that such benefits are available only to those who, by some biological norm, are supposed to have a uterus. The article that provides the 2013 update to the *Montreal Criteria* make this explicit, stating that UTx “offers the same promise of a solution for males and trans individuals wishing to gestate a child as it does for genetic females with UFI.”^2^ Nevertheless, they continue to require that “the recipient be a genetic female.”^3^

This article critically evaluates the *Montreal Criteria* (2012, 2013) as the primary ethical guidelines regarding UTx. This evaluation focuses on and challenges the way the *Criteria* structure the discussion around UFI as indication for UTx and the requirement for female genetics. I argue that, outside of the initial experimental design of UTx, there are *prima facie* grounds for making UTx available outside of the indication of UFI, and that for reasons both internal and external to the Montreal Criteria’s analysis of UTx, there are no overwriting ethical reasons to limit UTx to cis-gender women. Moving forward, ethical criteria for UTx should be more permissive, and be inclusive of individuals who are not assigned female at birth (nAFAB) but who may nevertheless benefit from UTx.

Ultimately, if we want to understand the limits of the diagnosis of UFI, then we must specify what it means to say that a person does not but *was supposed to have* a functioning uterus. And it’s not clear that “She was born with a uterus but required a hysterectomy” is a better answer to this query than “She was born without a uterus and assumed to be male at birth but has a female gender identity.” One might even suggest that the simple facts of infertility combined with the absence of a uterus is sufficient to constitute UFI, regardless of any facts around biological sex or gender.

## The Montreal Criteria

The issue regarding the ethical supportability of UTx has been most prominently addressed in “The Montreal Criteria for the Ethical Feasibility of Uterine Transplantation,” authored by Ariel Lefkowitz, Marcel Edwards, and Jacques Balayla (2012).^4^ The *Criteria* constitute an early framework for thinking about the ethics of UTx. As such, the document is looked to as the primary source for ethical guidance on this subject in the literature on this subject as well as by professional organizations. While there are currently no national or professional guidelines with respect to UTx, the International Society of Uterus Transplantation (ISUTx) is a likely candidate for establishing such guidelines, and their website lists the *Criteria* as a primary resource.^5^ This is to establish that the *Criteria* are significantly influential and will likely play a significant role in the future developments of UTx.

**Fig. 1:**
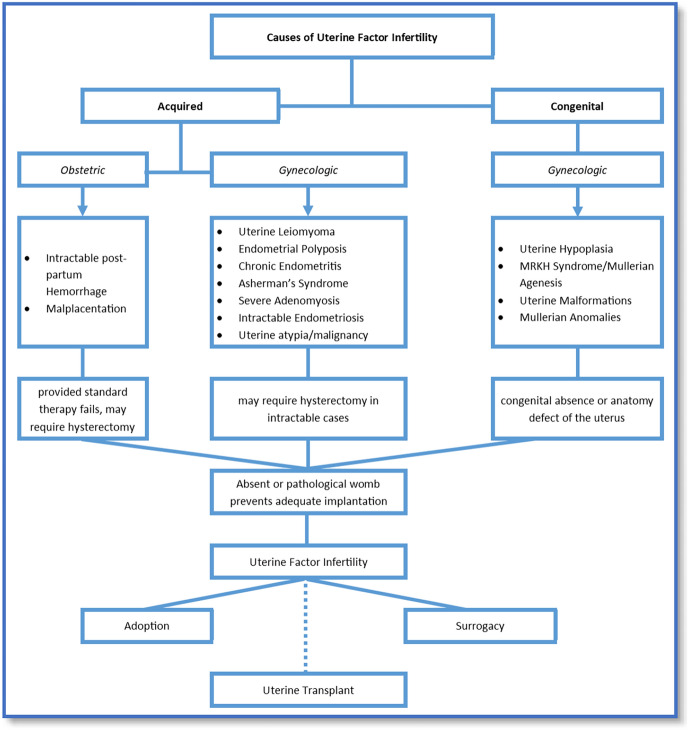
Causes of Uterine Factor Infertility (recreated from Lefkowitz, et al. (2012), p. 440)

The aim of the *Montreal Criteria* is to describe “the ethical issues in the context of experimentation and standard practice which surround this controversial and potentially paradigm-altering procedure” and to introduce “a set of proposed criteria required for a woman to be ethically considered a candidate for uterine transplant.”^6^ The *Criteria* spell out requirements for three relevant groups of stakeholders, namely the recipient, the donor, and the health care team. The primary focus throughout this article is on the requirements for potential UTx recipients, treating questions around donation and appropriate health care team requirements as distinct. One of the essential features of the *Criteria* is that it is clear about what UTx can offer and identifies the importance of the desire for the appropriate kind of experience of gestation. UTx is one of several options that exists with respect to family planning and reproduction. People who lack the ability to gestate have other family planning and reproductive options available to them, including being child free, adoption, and (depending on local law and regulation) surrogacy. Adoption can facilitate a family expansion if one is not primarily concerned with the experience of gestation or biological relationships. Surrogacy allows for family expansion without the experience of gestation, but while maintaining the biological relationship. UTx, like some other assisted reproductive options, allows for some of the experiences of gestation, whether or not the biological relationship is maintained.^7^ Figure 1 is a reproduction of the chart provided by Lefkowitz et al. to visualize the relationship between UFI and UTx.

UTx potentially enable the recipient to have a *limited* gestation experience. Lefkowitz et al. explain that “UTx does not offer women the opportunity *have* a baby, but rather to carry a pregnancy,” which “will not be an *ordinary* pregnancy” as the uterus “will not be innervated” which mean there will be no sensation of “fetal movements or contractions” and no possibility for “vaginal birth.”^8^ As it currently stands, the transplanted uterus is not enervated, and so the person carrying the pregnancy cannot feel fetal movements, and the uterus is unable to contract. And at this time, while the procedure can certainly evolve in this respect, currently vaginal delivery is not indicated in the context of UTx. This means that the transplant recipient is unable to experience vaginal delivery and must undergo delivery by C-section. Importantly, while parts of the literature on UTx take note of the possibility of the procedure addressing dysmorphia—not only by enabling some experience of gestation, but potentially also menstruation and the sensation of bodily wholeness or completeness—this potential benefit is significantly outweighed by the risks of requiring prolonged graft survival. UTx is generally intended to be temporary as increasing the longevity of the transplant past reproductive success sharply increases the associated risks.^9^ At this time, then, UTx is not indicated for the purpose of addressing body or gender dysmorphia, and this is currently not considered a benefit of the procedure.

Notably, the Montreal Criteria was updated by the original authors in “Ethical Considerations in the Era of the Uterine Transplant: An Update of the *Montreal Criteria for the Ethical Feasibility of Uterine Transplantation*” (2013).^10^ While this updated followed shortly after the original publication, the time in between was significant for UTx. Not only did this see an increase in human trials, but it also saw the publication of the Indianapolis Consensus.^11^ “Uterine Transplantation—A Real Possibility? The Indianapolis Consensus” was produced by Giuseppe Del Priore, Srdjan Saso, Eric Meslin, Andreas Tzakis, Mats Brännström, Alex Clarke, Rodrigo Vianna, Renata Sawyer, and Richard Smith (2013). The aim of the Consensus is to “present parameters that must be considered for UTn [UTx] to become an acceptable procedure in the human setting.”^12^ While the *Consensus* outlines some important goals and precautions for UTx research and the potential transition to clinical practice, the *Criteria* are far more detailed and influential. As a result, the focus remains on the latter.

**Fig. 2:**
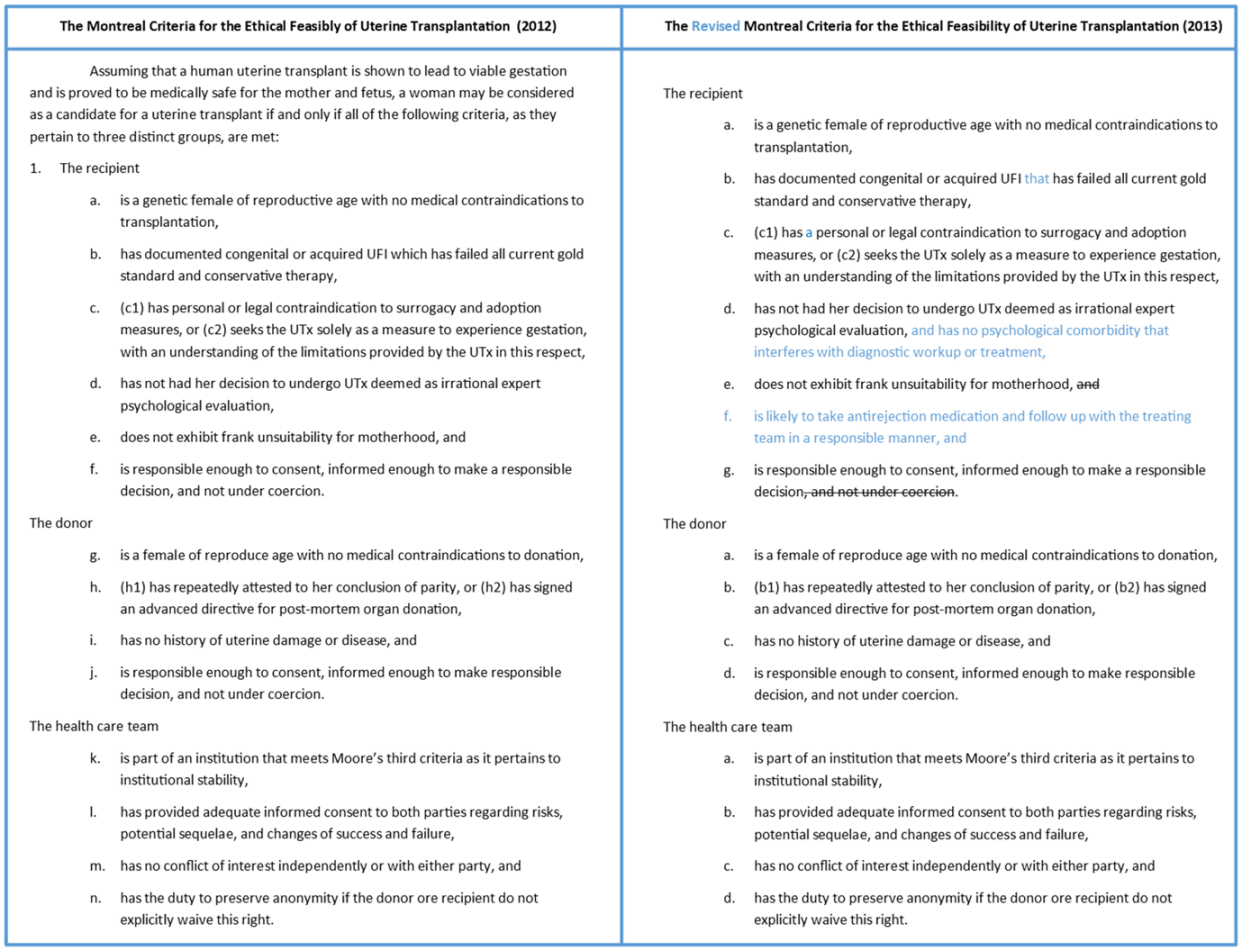
The Montreal Criteria for the Ethical Feasibility of Uterine Transplantation (recreated from Lefkowitz, et al. 2012, 445 and Lefkowitz, et al. 2013, 924)

The revisions in the updated *Criteria* itself are relatively minor. As evident in Fig. 2, besides making psychological contraindications and the ability to maintain a therapeutic relationship with the treatment team explicit features of the criteria, the revisions do not substantially affect the content of the *Criteria*. That said, the discussion contextualizing the update offers a valuable perspective on the advances and possibilities of UTx. Nevertheless, these considerations do not affect the content of the *Criteria*. This is an important shortcoming, because it is the *Criteria* as written that serve as a foundation for ethical considerations in the world of UTx. Moreover, while the update to the *Criteria* responded to a productive year, more than a decade has passed and UTx is making the transition from research to practice.^13^ This move opens up new possibilities, new opportunities for developing UTx in ways that were previously inaccessible. Navigating these possibilities requires a set of tools that is sufficiently forward-looking and adaptive. The next section will evident that despite the authors’ anticipating these changes, the *Criteria* as written are unable to sufficiently address them, and therefore stand in need of significant revision if UTx is to move into the future ethically and equitably.

## Evaluating the Montreal Criteria

The *Montreal Criteria* attempt to outline the ethical norms for UTx. They were produced at a time when research into UTx was still new and updated only shortly after. The *Criteria* are still broadly cited, are unable to meet the demands of a program that is moving into clinical practice in a social environment is changing with respect to how it understands gender. This remains true, despite 2013 update. As such, the criteria for the ethical supportability of UTx stand in need of significant update and revision. There are four primary issues with the *Montreal Criteria* that are relevant to the current analysis, namely:

1. an implicit commitment to UFI as the exclusive indication for UTx
2. an asymmetry between the requirements for potential recipients and donors
3. an inconsistent appeal to Moore’s ethical criteria for surgical innovation; and^14^
4. a commitment to biological essentialism.

Each of these features will be discussed in detail in this section, including whether and how the 2013 update addresses the issues.

### An implicit commitment to UFI as the exclusive indication for UTx

The first issue with the *Montreal Criteria* is that they identify uterine factor infertility (UFI) as the only potential indication for UTx. There are two major problems with identifying UFI as the single indication for UTx. First, this is based on an inference. The chart in Fig. 1, which indicates a dotted line between UFI and UTx, is the only pathway to UTx that is discussed in the *Criteria*. This functionally excludes anyone who is not a cis-gender woman or Assigned Female at Birth (AFAB) from consideration. The argument implicit in the discussion of the *Criteria* runs as follows:

Premise 1:If UFI, then possible UTx candidate.

Premise 2:No UFI.

Conclusion: No UTx candidate.

This argument form, namely denying the antecedent, constitutes a logical fallacy. Just because UFI is *an* indication for UTx candidacy does not entail that it is the *only* possible indication for UTx candidacy. Moreover, the chart in Fig. 1 is somewhat misleading in this respect, and the discussion provided in the article itself points to a different indication for UTx. The authors make explicit that UTx is not able to provide the experience of a “ordinary” pregnancy, and so insofar as a person is looking for such an experience, UTx is not a good fit. What is required is the “desire to experience gestation,” and more particularly a desire for the kind of gestational experience that UTx can offer.^15^ Moreover, that desire must be sufficiently strong to accept the risks that come with UTx, and therefore requires that the unmet desire for a gestational experience of the kind that UTx can offer is such that it impairs the person’s quality of life. In short, this means that what the *Criteria* describe is not, in fact, candidacy for UTx based on UFI, but rather candidacy based on a potential consequence of UFI, namely the impaired quality of life due to an inability to gestate a fetus. And whereas UFI is arguable limited to cisgender or AFAB women, this impaired quality of life is not.

Second, UFI appears to be definitionally limited to people who are biological female. There are several ways to define this category, for instance as being AFAB, as presenting with female neonatal genitalia, or having a XX karyotype. Furthermore, as evidenced by Fig. 2, the *Criteria* themselves require that the recipient is genetically female, possibly requiring a XX karyotype. Nevertheless, there are people who could be considered biologically female in a relevant way that is not appropriately captured this singular category. This issue will be further explored as part of the implicit commitment to gender essentialism.

These issues largely remain untouched in the 2013 update to the *Criteria* as written, though they are addressed in the discussion around the update. The authors insist that while UTx “offers the same promise of a solution for males and trans individuals wishing to gestate a child as it does for genetic females with UFI … the *Montreal Criteria* require that the recipient be a genetic female.” In this discussion of this, they acknowledge lack of a “*prima facie* ethical reason to reject the idea of performing uterine transplant on a male or trans person,” recognizing that a “male or trans patient wishing to gestate a child does not have a lesser claim to that desire than their female counterparts.” It is striking that this recognition does not meaningfully affect the guidelines themselves. Indeed, the revised *Criteria* continue to require that the recipient “is a genetic female,” that she has a “documented congenital or acquired UFI that has failed all current gold standard and conservative therapy,” and that she “does not exhibit frank unsuitability for motherhood.”^16^ The primary motivation for this appears to be a commitment to Moore’s surgical criteria, which will be discussed as part of #3.

### An asymmetry between the requirements for potential recipients and donors

The second issue concerns an asymmetry between recipient and donor requirements. It is notable that for the recipient, it is required that they are “a genetic female of reproductive age with no medical contraindication to transplantation.” By contrast, for the donor it is required that they are “a female of reproductive age with no medical contraindication to donation.” One notable thing here is that, where the recipients must be a “genetic female,” the donor must be a “female.” There are two straightforward interpretations of this. First, it may be the case that there is an implicit “genetic” in 2a. This would make the requirements symmetric and consistent. However, it would presumably exclude an AFAB person with XY karyotype who has Swyer syndrome from donating their uterus without any substantial justification for this barrier.

Second, it may be the case that the donor requirement is purposely ambiguous. It would therefore be permissible for an AFAB person with Swyer syndrome to donate their uterus, and the requirement would be inclusive of other potential genetic disorders that create ambiguity about the genetic sex of a person. One complication here is that, either the requirement would exclude transgender men from being able to donate their uterus, or the requirement misgenders transgender men. The former is prima facie unjustified. The latter requires that the Criteria are amended to address this issue. While this issue can be settled, it is evidence that there is an implicit commitment to biological essentialism underlying the Montreal Criteria. Assuming that there is no good justification to indiscriminately exclude a social group whose members generally have a uterus from uterus donation, we must instead accept that everyone with a uterus is a potential candidate for donation. In this case, since the recipient must be XX karyotype, there is a stark asymmetry between the requirements for recipients and donors. It violates a key principle of justice for a population to be able to donate into a pool to which that same population has no access.

### Inconsistent appeal to Moore’s ethical criteria for surgical innovation

The *Montreal Criteria* were developed in a particular historic context, at a time when UTx was in its infancy. Developing UTx in the context of clinical research required minimization of avoidable variables and sufficient collateral data around the innovative procedure to support its implementation in human subjects. The *Criteria* rely on Moore’s Criteria for Surgical Innovation (MCSI) to provide limits to the ethical acceptability of surgical innovation. Because Moore’s Criteria are baked into the guidelines for UTx, they are prerequisite for UTx to be ethically permissible. In other words, if UTx for AFAB patients did not have sufficient research background, then it would be unethical to proceed with UTx for AFAB patients. At the time, the authors tacitly supported that there was sufficient research background and collateral data to move forward with UTx as a surgical innovation for AFAB patients. The 2013 update to the *Criteria* were made in recognition of the rapid changes to the UTx research field, and the authors take note of the possibility of UTx for nAFAB patient. They even make the claim that there is *prima facie* obligation of justice to extend consideration for candidacy to nAFAB patients.^17^ Nevertheless, one of the primary reasons why this more permissive discussion fails to affect the *Criteria* as written, appears to be the authors’ conviction that expanding this expansion lacks an adequate research background.^18^

The problem here is two-fold. First, the judgment of whether or not the research background is sufficient is subjective and is possibly biased against expanding UTx. Given the current level of experience in successful UTx in humans, there arguably exists a more robust research background to justify broadening the scope of UTx than there existed before starting UTx in the first place. Second, neither the original *Criteria* nor the update as written can accommodate the changing landscape of UTx. The *Criteria* ought to be responsive to the growing body of evidence, including the successes of UTx for AFAB patients and recent animal studies.^19^ However, the *Criteria* as written *do not permit* the inclusion of nAFAB populations *even if* Moore’s Criteria are met, because their exclusion is otherwise stipulated. That is, as written, *even if UTx for nAFAB patients meets Moore’s Criteria, the Montreal Criteria entail that it would still be ethically unsupportable to proceed with UTx for nAFAB patients* (from 1a, b, and possibly e).

### Commitment to biological essentialism

As indicated in the previous section, some of the issues with the *Montreal Criteria* can be attributed to the historical conditions that saw their formulation. The procedure was under consideration specifically for cis-gender women, which from a research perspective makes a lot of sense. However, a lot of potential nuances have gotten lost in this process: a pragmatic focus on cisgender women has resulted in the exclusion of transgender women and other nAFAB persons from consideration, without the requisite justification. While the 2013 update to the *Criteria* discussed the possibility of a more inclusive approach to UTx, it failed to make good on this possibility in its revision. This ultimately presents us with the fourth major issue that has operated in the background of the exclusionary stance on UTx, i.e. the *Criteria*’s implicit commitment to some version of biological essentialism.

As evidenced by Fig. 2, the original and the updated *Criteria* both require a genetically female recipient. While a full ontological account of sex and gender is outside of the scope of this discussion, suffice it to say that is has been sufficiently demonstrated that biological sex is a complex phenomenon that produces exceptions to any attempt to formulate binary rules that attempt to define the categories of male and female, and that sex and gender are neither binary nor causally related.

Additionally, both the original and the updated *Criteria* require suitability for motherhood (per 1e), which the updated *Criteria* require in addition to a more general psychological evaluation and suitability (per 1d). It is unclear what suitability for motherhood adds to this. Motherhood can be understood in functional, biological, and social terms. Functional motherhood would refer to the capacity for gestation, in which case either (1) anyone with UFI is unsuitable for motherhood, or (2) anyone who is otherwise a good candidate for UTx is suitable for motherhood. In this case, the requirement adds nothing to the other criteria. Biological motherhood would reduce to the requirements in 1a, namely genetic and developmental suitability. If the requirement is to be additive, then, it must refer to some social conception of motherhood. In that case, more needs to be said about (1) what constitutes this type of motherhood and (2) why it should be normative in the context of UTx criteria. The risk of 1e is that it reinforces traditional notions of womanhood and motherhood as a requirement for gestating and having children. If the requirement is simply after a broad suitability to parenthood, then it should state that, specify what is required for such suitability, and defend any restriction of reproductive autonomy it entails.

The authors “emphasize that the *Montreal Criteria* are dynamic and subject to evolve with the advancement of medicine and the development of the transplant.”^20^ Moreover, they recognize that “A male who identifies as a woman … arguably has UFI, no functionally different than a woman who is born female with UFI.”^21^ These are important observations that are not scarcely elaborated on and, as discussed, do not affect the *Criteria*. As professional organizations and institutions look for resources to comment on UTx, they will inevitably find the *Montreal Criteria*. And whether they look to the original criteria or the 2013 update, they are likely to incorporate the *Criteria* as written, and as a result exclude nAFAB populations from consideration for UTx, significantly impacting their ability to access this care in the future. Therefore, the guidelines for ethical UTx stand in need of significant overhaul.

## The Montreal Criteria in the literature

The limits of the *Montreal Criteria* are to some degree recognized in the literature. Recent publications recognize the fact that the *Criteria* are not well suited for the current research environment. For instance, Ronchi and Napoletano noted that the *Criteria* “may itself be already outdated, in that it requires the recipient to be a “genetic female”, whereas research on the possibility to perform UTx on transgender women is already in progress.”^22^ And in “Uterus Transplantation and the Redefinition of Core Bioethics Precepts” (2021), Federica Ronchi and Gabriele Napoletano argue for the need of a new ethical framework for UTx. They state that “Current bioethics approaches need to undergo a radical update if we are to successfully meet the challenges posed by fast-growing scientific advances, set to shape and mold our lives ever more dramatically.”^23^ This, of course, does not entail that UTx is ultimately ethically justified, or that ethics must always endorse scientific advancements. However, it does not mean that our frameworks for ethical evaluation must be up to task of assessing new procedures, treatments, methodologies, and so on. These considerations demonstrate that, rather than look at the *Montreal Criteria* as definitive, we ought to see it as a product of its time and place and acknowledge its social and historical limitations. At the same time, we can recognize the Criteria’s vital contribution to the ethical discussion concerning UTx. It is in identifying both the strengths and weakness of the Criteria that they can serve as a steppingstone toward a more inclusive framework for UTx.

## Barriers to moving beyond montreal

There may be barriers to expanding indication for UTx to nAFAB individuals. General surgical complexity is no doubt compounded in the setting of introducing a new organ to the body. And this difficulty may be further excecated if prior surgeries have introduced scar tissue in the abdomen.^24^ Some of these issues have been considered in the literature, suggesting that there is anatomical compatibility without “overwhelming clinical argument *against* performing UTx as part of” gender affirming surgery.^25^ Early research is also underway, with evidence of surgical feasibility animal models.^26^ While more research needs to be done to assess the surgical feasibility of uterine transplant in nAFAB individuals, this does not necessitate a pause on the ethical work regarding the same.

## Conclusion

In medicine, ethical frameworks are often part of the essential scaffolding of research and innovation. Ethical guidelines specify conditions for permissibility out of which practice develops. Frequently, the constraints entailed by these guidelines are meant to ensure safe and informed processes, appropriately balanced potential for harms and benefits, and prioritization of the aim to protect vulnerable populations. However, excess constraints can produce contrary results. In the case of UTx, limiting considerations to cis-gender or AFAB women with UFI excludes already vulnerable populations from accessing a unique kind of care that has the potential to address a similar, if not identical, desire. The ethics of expanding UTx candidacy to nAFAB individuals is, of course, contingent. It assumes that UTx is an ethically supportable practice with an appropriate risk-benefit profile, that uterine donation is ethically permissible, that UTx is physiologically possible for nAFAB individuals, and that there are no other potential harms that outweigh the anticipated benefits. That said, given that the nAFAB population that would be best served by an expansion of UTx would include transgender women, a historically and currently significantly disenfranchised minority population, this adds pressure to investigate the feasibility of UTx in nAFAB individuals and developing a coherent ethical framework that can accommodate a more inclusive approach to UTx.

## Data Availability

No datasets were generated or analysed during the current study.

## Footnotes

1 The case of Lili Elbe, a Danish transgender woman living in France, who underwent a series of gender affirming operations, including uterus transplantation, around 1930. “She died of complications not long after the fifth procedure in 1931.” “Lili Elbe | Biography, Wife, Art, Surgeries, & Facts | Britannica.” (2024).

2 Lefkowitz et al., “Ethical Considerations in the Era of the Uterine Transplant.”

3 Lefkowitz et al., “The Montreal Criteria for the Ethical Feasibility of Uterine Transplantation.”

4 Lefkowitz et al., “The Montreal Criteria for the Ethical Feasibility of Uterine Transplantation.”

5 Horvat and Iltis (2019), “What Are Good Guidelines for Evaluating Uterus Transplantation?,” 989.

6 Lefkowitz et al., “The Montreal Criteria for the Ethical Feasibility of Uterine Transplantation,” 439.

7 Lefkowitz et al., “The Montreal Criteria for the Ethical Feasibility of Uterine Transplantation,” 440, 445.

8 Lefkowitz et al., “The Montreal Criteria for the Ethical Feasibility of Uterine Transplantation,” 442.

9 Jones et al. (2021), “Uterine Transplantation in 2021.” Jones et al., “Perceptions and Motivations for Uterus Transplant in Transgender Women.”

10 Lefkowitz et al., “Ethical Considerations in the Era of the Uterine Transplant.”

11 Lefkowitz et al., “Ethical Considerations in the Era of the Uterine Transplant,” 924.

12 Del Priori et al., “Uterine Transplantation—a Real Possibility? The Indianapolis Consensus,” 228.

13 Brännström et al. (2023), “Uterus Transplantation.”

14 Moore (2000), “Ethical Problems Special to Surgery.” Morgenstern (2006), “Innovative Surgery’s Dilemma.”

15 Lefkowitz et al., “The Montreal Criteria for the Ethical Feasibility of Uterine Transplantation.”

16 Lefkowitz et al., “Ethical Considerations in the Era of the Uterine Transplant.”

17 Lefkowitz et al., “Ethical Considerations in the Era of the Uterine Transplant.”

18 Lefkowitz et al., “Ethical Considerations in the Era of the Uterine Transplant.”

19 Tay, “Study That Impregnated Male Rats Stirs Controversy.”

20 Lefkowitz et al., “Ethical Considerations in the Era of the Uterine Transplant.”

21 Lefkowitz et al., “Ethical Considerations in the Era of the Uterine Transplant.”

22 Umani Ronchi and Napoletano (2021), “Uterus Transplantation and the Redefinition of Core Bioethics Precepts,” 1.

23 Umani Ronchi and Napoletano (2021), “Uterus Transplantation and the Redefinition of Core Bioethics Precepts,” 2.

24 Thanks to the reviewers for raising this issue and providing this citation; Panagiotakopoulos et al. (2020), “The Effect of Early Puberty Suppression on Treatment Options and Outcomes in Transgender Patients.”

25 Jones et al. (2022), “Perceptions and Motivations for Uterus Transplant in Transgender Women,” 155.

26 Tay (2021), “Study That Impregnated Male Rats Stirs Controversy.” These issues, along with suggestions for revisions of the Montreal Criteria, are discussed in more detail in Pannekoek (Forthcoming), “Beyond Cis-Gender Women: Ethical Recommendations Regarding Uterine Transplantation for Transgender Women and nAFAB Persons.”
